# Evolution of the gut microbiome following acute HIV-1 infection

**DOI:** 10.1186/s40168-019-0687-5

**Published:** 2019-05-11

**Authors:** Muntsa Rocafort, Marc Noguera-Julian, Javier Rivera, Lucía Pastor, Yolanda Guillén, Jost Langhorst, Mariona Parera, Inacio Mandomando, Jorge Carrillo, Víctor Urrea, Cristina Rodríguez, Maria Casadellà, Maria Luz Calle, Bonaventura Clotet, Julià Blanco, Denise Naniche, Roger Paredes

**Affiliations:** 1 IrsiCaixa AIDS Research Institute, Ctra de Canyet s/n, 08916 Badalona, Catalonia Spain; 2 Universitat Autònoma de Barcelona, 08193 Bellaterra, Catalonia Spain; 3 Universitat de Vic-Universitat Central de Catalunya, C. Sagrada Família 7, 08500 Vic, Catalonia Spain; 4 ISGlobal, Barcelona Centre for International Health Research (CRESIB), Hospital Clínic–Universitat de Barcelona, 08036 Barcelona, Catalonia Spain; 5 Institut Germans Trias i Pujol (IGTP), Hospital Germans Trias i Pujol, Universitat Autonoma de Barcelona, 08916 Badalona, Catalonia Spain; 6 Centro de Investigação em Saúde da Manhiça (CISM), 1929 Maputo, Mozambique; 7 Integrative Gastroenterology, Kliniken Essen-Mitte, University of Duisburg-Essen, Essen, Germany; 8 Chair for Integrative Medicine and translationale Gastroenterology, Klinikum Bamberg, University of Duisburg-Essen, Essen, Bavaria Germany; 9 Infectious Diseases Service, Hospital Universitari Germans Trias i Pujol, Ctra de Canyet s/n, 08916 Badalona, Catalonia Spain

**Keywords:** Microbiome, HIV-1, acute HIV-1 infection, HIV-1 pathogenesis, AIDS

## Abstract

**Background:**

In rhesus macaques, simian immunodeficiency virus infection is followed by expansion of enteric viruses but has a limited impact on the gut bacteriome. To understand the longitudinal effects of HIV-1 infection on the human gut microbiota, we prospectively followed 49 Mozambican subjects diagnosed with recent HIV-1 infection (RHI) and 54 HIV-1-negative controls for 9–18 months and compared them with 98 chronically HIV-1-infected subjects treated with antiretrovirals (*n* = 27) or not (*n* = 71).

**Results:**

We show that RHI is followed by increased fecal adenovirus shedding, which persists during chronic HIV-1 infection and does not resolve with ART. Recent HIV-1 infection is also followed by transient non-HIV-specific changes in the gut bacterial richness and composition. Despite early resilience to change, an HIV-1-specific signature in the gut bacteriome—featuring depletion of *Akkermansia*, *Anaerovibrio*, *Bifidobacterium*, and *Clostridium—*previously associated with chronic inflammation, CD8+ T cell anergy, and metabolic disorders, can be eventually identified in chronically HIV-1-infected subjects.

**Conclusions:**

Recent HIV-1 infection is associated with increased fecal shedding of eukaryotic viruses, transient loss of bacterial taxonomic richness, and long-term reductions in microbial gene richness. An HIV-1-associated microbiome signature only becomes evident in chronically HIV-1-infected subjects.

**Electronic supplementary material:**

The online version of this article (10.1186/s40168-019-0687-5) contains supplementary material, which is available to authorized users.

## Background

There is cumulative evidence that the gut microbiota plays an important role in HIV-1 pathogenesis. The gut-associated lymphoid (GALT) and epithelial tissues are severely and rapidly damaged following HIV-1 infection [1]. The resulting local and systemic inflammation and loss of CD4^+^ T cells, chronic immune activation, and immune deregulation [2–9] are not fully restored with antiretroviral therapy (ART). Chronic inflammation, immune activation, and endotoxemia are linked to the precocious development of aging-related diseases like type 2 diabetes, cardiovascular diseases, and frailty syndrome in people living with HIV (PLWH) [3, 10–12]. All such disorders have been associated with gut microbiota alterations in non-HIV-infected subjects [13–15]. It is thus conceivable that the gut microbiome might also be involved in the premature aging of PLWH.

However, the precise mechanisms governing the interplay between the host immune system and HIV-1, as well as the exact changes occurring in the gut microbiome following HIV-1 infection, remain to be defined. Cross-sectional studies consistently show reduced bacterial diversity and enrichment in *Proteobacteria* in PLWH, which are linked to lower CD4^+^ T cell counts [16], higher inflammation, and increased immune activation [5, 17–19]. Previously reported associations between *Prevotella* predominance and HIV-1 infection have been shown to be confounded by HIV-1 risk group [19–22]. Using fecal shotgun metagenomics, we previously found that nadir CD4+ T cell count, the main predictor of clinical complications in PLWH, is also a major independent predictor of reduced microbial gene richness and gut microbial shifts in this population [23]. Such shifts include depletion of methanogens, sulfate-reducing bacteria, and other oxygen-sensitive syntrophic microbes, coupled with enrichment in ROS/RNS-resistant microbes like *Bacteroides* and *Proteobacteria*.

Longitudinal studies in non-human primates, which allow to control for confounders affecting human studies have consistently reported increases in taxa from phylum *Proteobacteria*, such as *Actinobacillus* spp. and *Aggregatibacter* spp., as well as in potential pathogens from the *Mycoplasmacetae* family and *Staphylococcus spp.* following simian immunodeficiency virus (SIV) infection [24, 25]. However, no consistent changes are observed in other major phyla like *Bacteroidetes* or *Firmicutes*, suggesting that gut microbiome changes after SIV infection are pleiotropic [24]. Furthermore, in non-human primate models, pathogenic but not non-pathogenic SIV infection is linked to an expansion of the enteric virome, including *Adenovirus*, *Picornavirus*, and *Anellovirus*, which is associated with gut epithelial damage, tissue inflammation, and gut leakiness [26].

In the only longitudinal study available to date in humans [27], the gut microbiome of 59 subjects diagnosed with HIV-1 infection during Fiebig I–IV stages was enriched in phylum *Bacteroidetes* and depleted in phyla *Firmicutes* and *Proteobacteria* compared to 26 HIV-negative controls. Antiretroviral treatment (ART) initiation was associated with relative increases in *Fusobacteria*, *Proteobacteria*, and *Tenericutes* and decreases in *Bacteroidetes* and *Firmicutes*. Such findings are in contrast with non-human primate data [24, 25] which report opposite dynamics regarding *Proteobacteria*, as well as with data from cross-sectional human studies [5, 18, 28, 29]. Moreover, they provide limited information below the phylum taxonomic level, which is essential to understand the pathophysiology of gut dysbiosis in HIV infection and to devise future interventions on the gut microbiome. The initiation of ART shortly after diagnosis did not enable a more prolonged evaluation of the gut microbiome dynamics following HIV-1 infection. Finally, the study did not provide insights as to the role of intestinal virus expansion after HIV-1 infection.

Here, we present the results of a prospective and controlled cohort study investigating the evolution of the gut bacteriome and of several clinically relevant eukaryotic viruses following HIV-1 infection in Mozambique. Longitudinal microbiome data are compared with cross-sectional samples from treated and non-treated chronically HIV-1-infected subjects from the same setting. Gut microbiome findings are analyzed in the context of extensive clinical and microbiological metadata to rule out potential confounders and are associated with a comprehensive panel of immune markers, including inflammation, activation, gut barrier integrity, and bacterial translocation. Finally, a whole metagenomic analysis is performed on a subset of patients to gain further insights into gut microbial species and functional dynamics following HIV-1 infection.

## Results

### Study subjects

Out of 4011 subjects screened for fever-like illness or undergoing voluntary HIV-1 testing in the Manhiça District Hospital, Mozambique, 85 (2.1%) fulfilled criteria for recent HIV-1 infection (RHI). Forty-nine of them plus 55 HIV-negative (NEG) individuals consented to participate in this microbiome study (Additional file 1: Table S1). In addition, 98 chronically HIV-1-infected subjects, 27 of them receiving ART (CHI_ART), mainly consisting on NVP, 3TC and AZT, and 71 ART-naive (CHI_noART), provided a single fecal and blood sample for cross-sectional comparisons with RHI and NEG (Additional file 1: Figure S1). For such cross-sectional comparison, the longitudinal follow-up of RHI was divided in two periods: before and after the first 6 months of follow-up (RHI ≤ 6 and RHI > 6), and only data from the first sample available from each period, as well as the first sample available from each NEG subject, were included.

At the first study visit (baseline, Table 1) chronically HIV-1-infected subjects were older, had slightly higher BMI scores, and were less likely to have suffered malaria in the previous month than the remaining participants (Table 1). As expected, compared with HIV-negative individuals, RHI subjects had lower CD4+ T cell, higher CD8+ T cell, and lower platelet counts. There were no significant differences between groups in the prevalence of hepatitis B virus, syphilis, and multiple other microbial determinations. Longitudinally, the RHI group showed statistically significant decreases in CD8^+^ T cell counts and viral load, together with a significant increase in CD4^+^/CD8^+^ ratio, during the first 6 months after HIV-1 acquisition (Additional file 1: Figure S2).

**Table 1:** Baseline subject’s characteristics

		Recent HIV-1 infection	Chronic HIV-1 infection		HIV-Negative	*p* value	
			ART-naive	On ART		RHI vs neg	Overall
N = 202		49	71	27	55		
Female gender		32 (65%)	52 (73%)	15 (55%)	43 (78%)	0.189	0.154
Age (years)		26 (20; 30)	35 (29; 45.5)	42 (36.5; 46.5)	25 (21; 37)	0.210	< 0.001
Weight (kg)		55 (49; 62)	63 (53.5; 68.5)	61 (60; 68.5)	58.5 (52; 67)	0.093	0.007
Height (cm)		160 (155; 169.7)	160 (154.2; 164)	164 (157.5; 167.5)	163.9 (160; 170.8)	0.192	0.025
BMI		21 (19.1; 23.5)	23.7 (21; 27.6)	23.3 (21.5; 25)	22.2 (18.5; 25)	0.637	< 0.001
Pregnancy		3 (6.1%)	0	0	7 (12.7%)	0.730	0.003
CD4^+^ T cell/mm^3^		572 (409; 677)	533 (430.5; 726)	460 (366; 594.5)	928 (741; 1142.8)	< 0.001	< 0.001
CD8^+^ T cell/mm^3^		1229 (740; 1683)	988 (763.5; 1304)	834 (657.5; 1130)	591 (387.5; 689.7)	< 0.001	< 0.001
CD4^+^/CD8^+^		0.42 (0.23; 0.70)	0.56 (0.38; 0.79)	0.52 (0.33; 0.88)	1.65 (1.21; 2.24)	< 0.001	< 0.001
HIV-1 RNA at timepoint 1 (copies/mL)		108,220 (31,898; 273,650)	33,400 (7288; 105,200)	75 (75; 272.5)	–	–	< 0.001
HIV-1 RNA at screening (copies/ml)		2,752,700 (179,220; 15,078,000)	–	–	–	–	–
Time since HIV-1-diagnosis, days		–	1344 (977; 1737)	1814 (1463; 2302)	–	–	0.018
Time on ART, days		–	–	1257 (678; 1640)	–	–	–
Fiebig stage at screening*	III	24 (49%)	–	–		–	–
	IV	7 (14.2%)	–	–		–	–
	V	7 (14.2%)	–	–		–	–
	VI	11 (22.6%)	–	–		–	–
White blood cells		5.8 (4.4; 6.7)	5.2 (4.2; 6.4)	3.7 (3.2; 5)	5.2 (4.4; 6.5)	0.413	< 0.001
Hemoglobin		12.1 (10.9; 13.1)	11.8 (10.4; 12.7)	11.4 (10.8; 12.7)	12.3 (11.6; 13.2)	0.409	0.114
Hematocrite		36.9 (33.5; 39.8)	35.6 (32.7; 38.5)	34.4 (33; 38)	37.7 (34.7; 40.1)	0.320	0.044
Platelets		184.5 (140.7; 230.7)	205 (174.5; 258)	192 (173.5; 216)	226 (185; 265.5)	0.004	0.028
Hepatitis B		4 (8.1%)	2 (2.8%)	3 (11.1%)	1 (1.8%)	0.173	0.125
Syphilis		2 (4.1%)	0	1 (3.7%)	4 (7.3%)	0.682	0.089
Diarrhea, previous week		7 (14.3%)	3 (4.2%)	1 (3.7%)	4 (7.3%)	0.343	0.214
Temperature (°C)		36.3 (36.2; 36.5)	36.4 (36.2; 36.4)	36.4 (36.2; 36.4)	36.2 (36.1; 36.5)	0.956	0.509
Fever, previous 24 h		7 (14.3%)	3 (4.2%)	1 (3.7%)	3 (5.4%)	0.186	0.186
Ritchie test		2 (4.1%)	2 (2.8%)	0	1 (1.81%)	0.600	0.864
*Entamoeba hystolitica*		2 (4.1%)	6 (8.4%)	1 (3.7%)	2 (3.6%)	1	0.733
*Giardia* spp		2 (4.1%)	2 (2.8%)	0	5 (9.1%)	0.438	0.236
*Cryptosporidium* spp		0	2 (2.8%)	0	0	1	0.635
*Clostridium difficile*		2 (4.1%)	0	1 (3.7%)	1 (1.8%)	0.602	0.458
*Strongyloides*		1 (2.0%)	0	0	1 (1.8%)	1	0.636
Malaria, prev. month		7 (14.3%)	1 (1.4%)	0	9 (16.4%)	1	< 0.001
Current malaria test		2 (4.1%)	1 (1.4%)	0	0	–	0.071
Malaria test severity		3 (1.5; 3)	4 (4; 4)	NA	NA	–	0.071

Differences in continuous variables were evaluated using ANOVA test (except for the time since HIV-1 diagnosis, which is evaluated using a Student *T* test). Differences in categorical variables were tested using the Fisher's test. The statistical significance threshold was set to *P* = 0.05

*Fiebig stage determination at screening visit is described in [30]. Malaria test was only performed in subjects reporting febrile symptoms. Malaria severity is measured in a scale of 1 to 5, from light to severe

### 16S rRNA gene sequencing

#### Alpha diversity

**Fig. 1:**
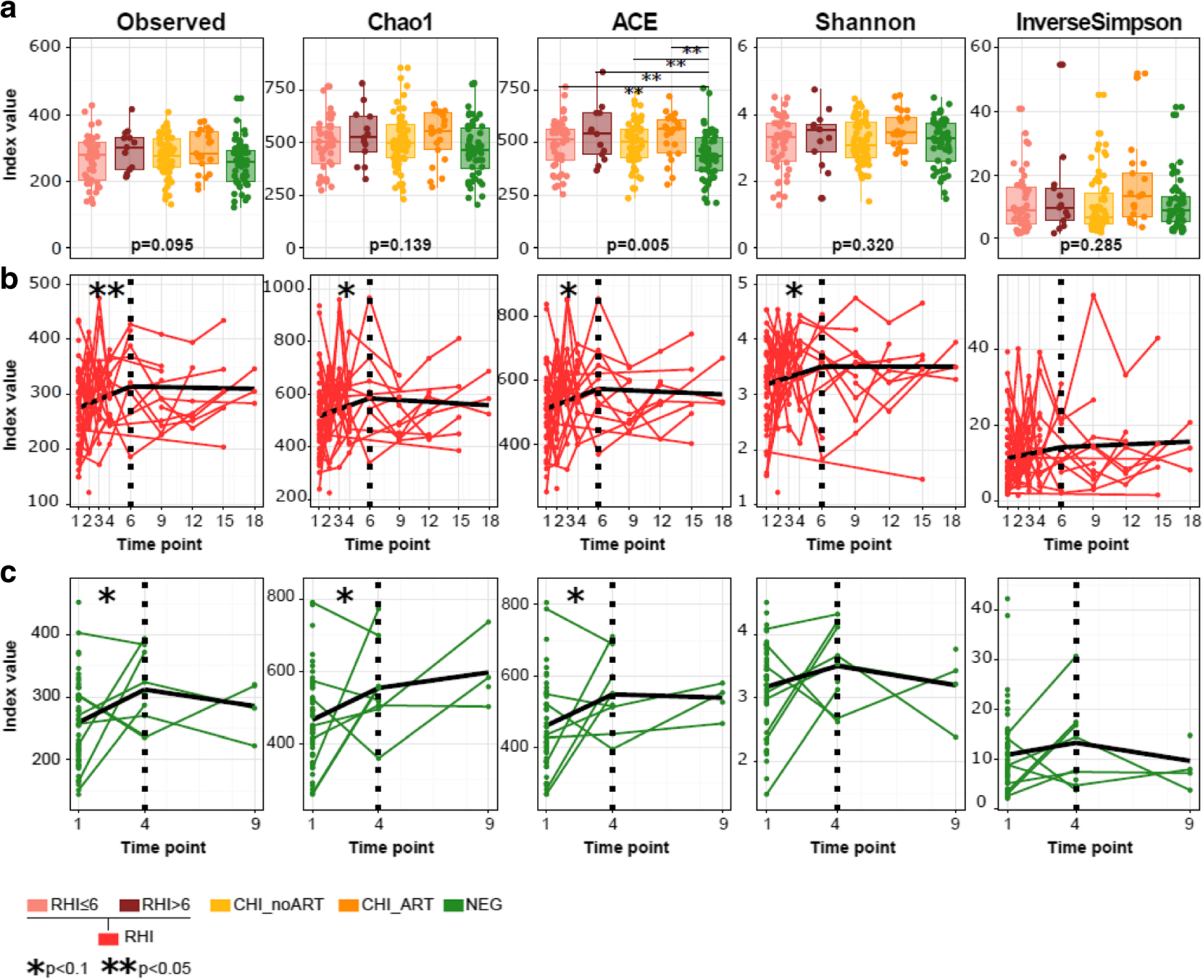
Microbial genus richness and diversity indices obtained with 16S rRNA gene sequencing. **a** Median ± IQR values in different cross-sectional comparison groups. Kruskal–Wallis *p* values are shown at the bottom of each plot. Asterisks (*p* values *< 0.1 and **< 0.05) highlight statistically significant Tukey post hoc pairwise differences between groups, corrected for multiple comparisons (FDR < 0.05). **b**, **c** Linear mixed models of the dynamics of richness and diversity indices in recently HIV-infected (RHI, red) and HIV-negative (NEG, green) subjects over time. Horizontal axes show months after study enrollment. Each dot corresponds to a sample, and samples from the same individual through follow-up are line-connected. Single dots correspond to individuals with no longitudinal follow-up. Thick black lines correspond to the modeled slope of each parameter. Vertical dashed lines show the inflection point at month 6 used for modelling in **b** and **c**. Statistically, significant differences from 0 (flat slope) are shown with asterisks. *p* values *< 0.1 and **< 0.05

Genus richness and diversity were not different between cross-sectional comparison groups except for the ACE richness numeric parameter, which was significantly lower in HIV-negative subjects than in the remaining groups (Fig. 1a). Longitudinally, richness and diversity increased during the first 6 and 4 months in both RHI and NEG subjects, respectively (Fig. 1b), with similar slopes between the two groups. Only gender was significantly associated to differences in microbial richness and diversity considering the baseline dataset, with men showing significantly lower values than female. Longitudinally, men showed stronger increases along follow-up compared to females (data not shown).

#### Microbial genus composition

Using non-metric multi-dimensional scaling (NMDS) on microbial phyla and genera composition data matrices, there were no significant clustering patterns between cross-sectional comparison groups, regardless of the distance metric used (Additional file 1: Figure S4). Partition around medoids (PAM) algorithm did not support microbiome clustering, and none of the metadata variables showed a significant differential effect on gut microbiome composition groups in PERMANOVA analyses (not shown). However, there was a statistically significant reduction in genus beta-diversity from RHI < 6 to RHI > 6 (Additional file 1: Figure S5).

**Fig. 2:**
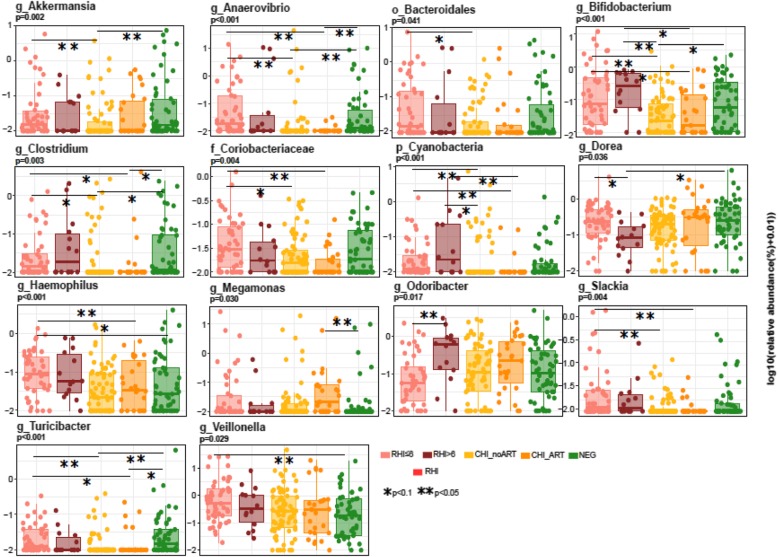
Differences in bacterial genera between groups. Box plots show median (± IQR) abundance of bacterial genera. Bacterial genera named “unclassified” are identified by their closest taxonomic level identification. Only bacterial genera with a significantly different abundance between groups (Kruskal–Wallis *p* value < 0.05) are shown. Statistically significant post hoc pairwise differences (Tukey post hoc pairwise tests corrected for multiple comparisons, FDR < 0.05) are shown with asterisks. Only the first microbiome measurement obtained RHI < 6, RHI > 6, and NEG was used for cross-sectional comparisons with CHI_ART and CHI_noART

In cross-sectional group comparisons (Fig. 2), CHI_noART were significantly depleted in *Akkermansia*, *Anaerovibrio*, *Bifidobacterium*, and *Clostridium*, relative to NEG individuals. Antiretroviral treatment exposure (CHI_ART) was not associated with changes in abundance of such genera, relative to CHI_noART. Individuals with RHI < 6 months were enriched in *Haemophilus* and *Veillonella* relative to HIV-negative subjects*.* Finally, there was an enrichment in *Odoribacter* and depletion in *Dorea* in RHI > 6 months relative to RHI < 6 months.

**Fig. 3:**
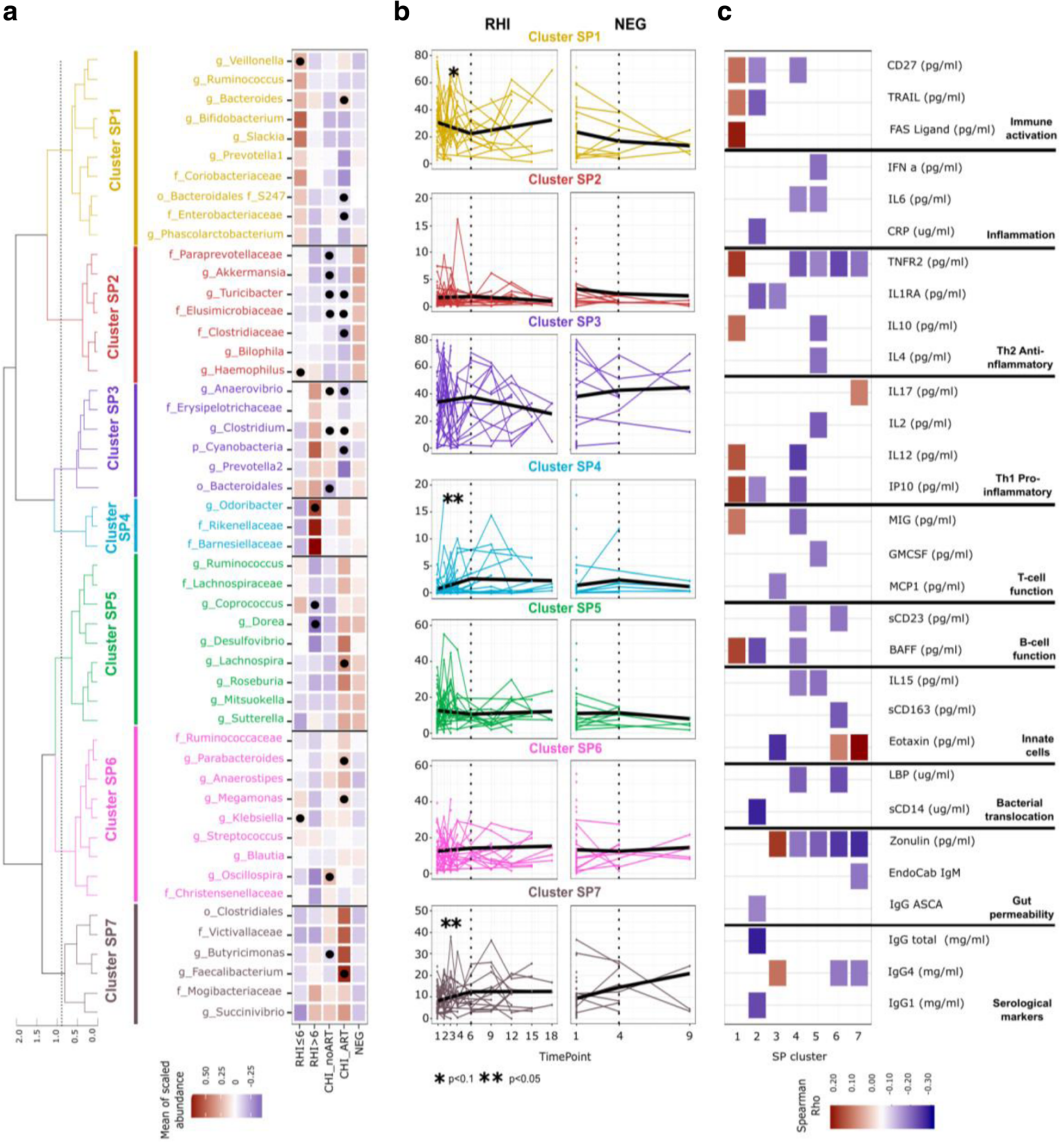
Dynamics of bacterial clusters following HIV-1 infection. **a** Within group co-abundant bacterial genus clusters (SP1 to 7) obtained using 16S rRNA gene sequencing. The color gradient is proportional to the mean of scaled individual relative abundance values (mean = 0, sd = 1) per bacterial genera and study group. Dots show statistically significant differences in genus abundance relative to HIV-negative subjects (NEG). b Linear mixed models of the longitudinal evolution of bacterial clusters in subjects with recent HIV-1 infection (RHI) and HIV-negative (NEG) individuals. Horizontal axes show months after study enrollment. Each dot corresponds to a sample, and samples from the same individual through follow-up are line-connected. Single dots correspond to individuals with no longitudinal follow-up. Thick black lines correspond to the modeled slope of each bacterial cluster. Statistically significant differences from 0 (flat slope) are shown with asterisks. *p* values *< 0.1 and **< 0.05. c Spearman’s correlation between bacterial clusters and immune markers measured in blood. The color gradient is proportional to the Spearman’s rho value. Only unadjusted statistically significant correlations (*p* value < 0.05) are shown. CHI_ART, CHI_noART, and first available samples from individuals in any of the RHI < 6, RHI > 6, and NEG groups were used to compute correlation values. Other immune markers measured in blood include IgA, IgM, IgG2, and IgG4 for serological makers; EndoCab IgG and IgA ASCA for gut permeability; FABP2 for bacterial translocation; IL7, IL13, GCSF, RANTES, MIP1 alpha, and beta for T cell function; IFN gamma, TNF alpha, and IL8 for Th1 pro-inflammatory responses; TGF beta for anti-inflammatory responses; CD40 ligand and IL21 for B cell function, Eotaxin, IL5, sCD163, and IL15 for innate cells; CXCL16 and IL1 beta for inflammation; B7H1, PDL2, and IL2R for immune activation; and EGF and VEGF for angiogenesis. Several markers were also measured in feces although none of them showed significant correlations with bacterial clusters: sIgA, ANCA, and ASCA for serological markers; EDNEPX, calprotectin, PMNE, lactoferrin, and S100A12 for neutrophil and eosinophil activation; and HBD2, zonulin, and alpha 1 antitrypsin for enterocyte damage and gut permeability. No correlations were found between bacterial clusters and levels of CD4^+^ and CD8^+^ T cell activation, exhaustion, and senescence in blood.

To evaluate the longitudinal evolution of microbial genera, 7 coabundant microbial genus clusters were identified (SP1 to 7, Fig. 3a, b). Only clusters SP1, 4, and 7 showed significant longitudinal changes during follow-up, and only in subjects with RHI (Fig. 3b). Cluster SP1, which was associated with increased plasma levels of cytokines involved in TNF-mediated innate responses (IL-10, IL-12, IP-10, BAFF, CD27, Fas ligand, and TNF receptor 2), decreased during the first 6 months of follow-up (Fig. 3c). Conversely, clusters SP4 and SP7, which included *Odoribacter*, *Rikenellaceae* unclassified, and *Barnesiaellaceae* unclassified and *Clostridiales* unclassified, *Butyricimonas*, *Faecalibacterium*, and *Succinivibrio*, respectively, increased during the same period (Fig. 3b). There was no evidence, however, that the longitudinal evolution of such clusters was significantly different between RHI and NEG. Clusters SP2 and SP3 did not significantly change in abundance over time, but confirmed the aforementioned observations regarding reduced *Akkermansia*, *Anaerovibrio*, and *Clostridium* in CHI_noART, relative to HIV-negative individuals. Moreover, SP2 was negatively correlated with markers of microbial translocation (sCD14), inflammation (IP10), and gut integrity (IgG ASCA) (Fig. 3c).

### Whole shotgun metagenome sequencing

#### Alpha diversity

Shotgun sequencing data also showed increasing trends in microbial richness and Shannon diversity over time (Additional file 1: Figure S3) in both RHI and NEG groups, suggesting that such changes in ecological parameters were not HIV-1-specific. Differences were not statistically significant, however, due to limited power.

**Fig. 4:**
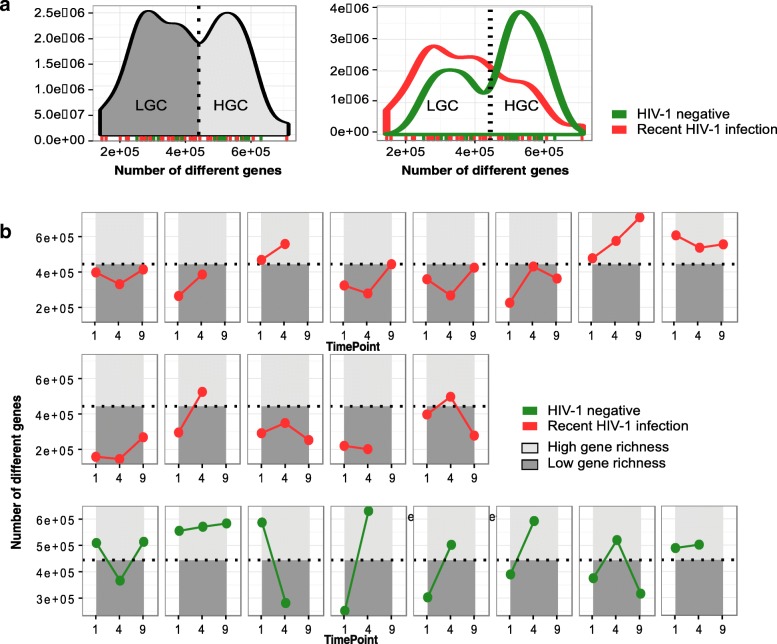
Microbial gene richness in recently HIV-1-infected and HIV-negative subjects using shotgun metagenomics. **a** The leftmost density plot shows a bimodal distribution of all samples according to their observed gene richness value, which enables their classification into low (LGC) and high gene count (HGC). The rightmost density plot shows that HGCs are enriched in NEG, whereas RHI predominate in LGCs. **b** Longitudinal evolution of microbial gene richness in RHI (in red) and NEG (in green). Each box represents an individual with its longitudinal follow-up samples (timepoints 1, 4, and 9). The dark gray-colored area represents the LGC zone, whereas light gray-colored area represents the HGC zone

Previous publications in European subjects have linked low values of gut microbial gene richness to metabolic dysregulation, obesity, immune activation, and inflammation [13, 31, 32]. In agreement with previous reports, the gut microbial gene richness also followed a bimodal distribution in Mozambican individuals. Samples with gene counts above and below a gene count threshold of 444.219 genes were categorized as high (HGC) and low gene counts (LGCs), respectively, (Fig. 4). Whereas 63% (22/35) of the RHI samples were included in the LGC category, only 37% (7/19) of HIV-negative samples were LGC (Fisher’s test *p* value 0.003) (Fig. 4a). At the first timepoint, 10/13 (76.9%) of RHI vs 4/8 (50%) of HIV-negative subjects (Fisher’s test *p* value 0.172) were already LGC (Fig. 4b). Eight of 13 (61.5%) subjects from the RHI group, but none of the HIV-negative individuals showed low gene count values in all follow-up timepoints. Moreover, 5/8 HIV-negative subjects (62.5%) showed increases in gene richness during follow-up.

#### Microbial species composition

**Fig. 5:**
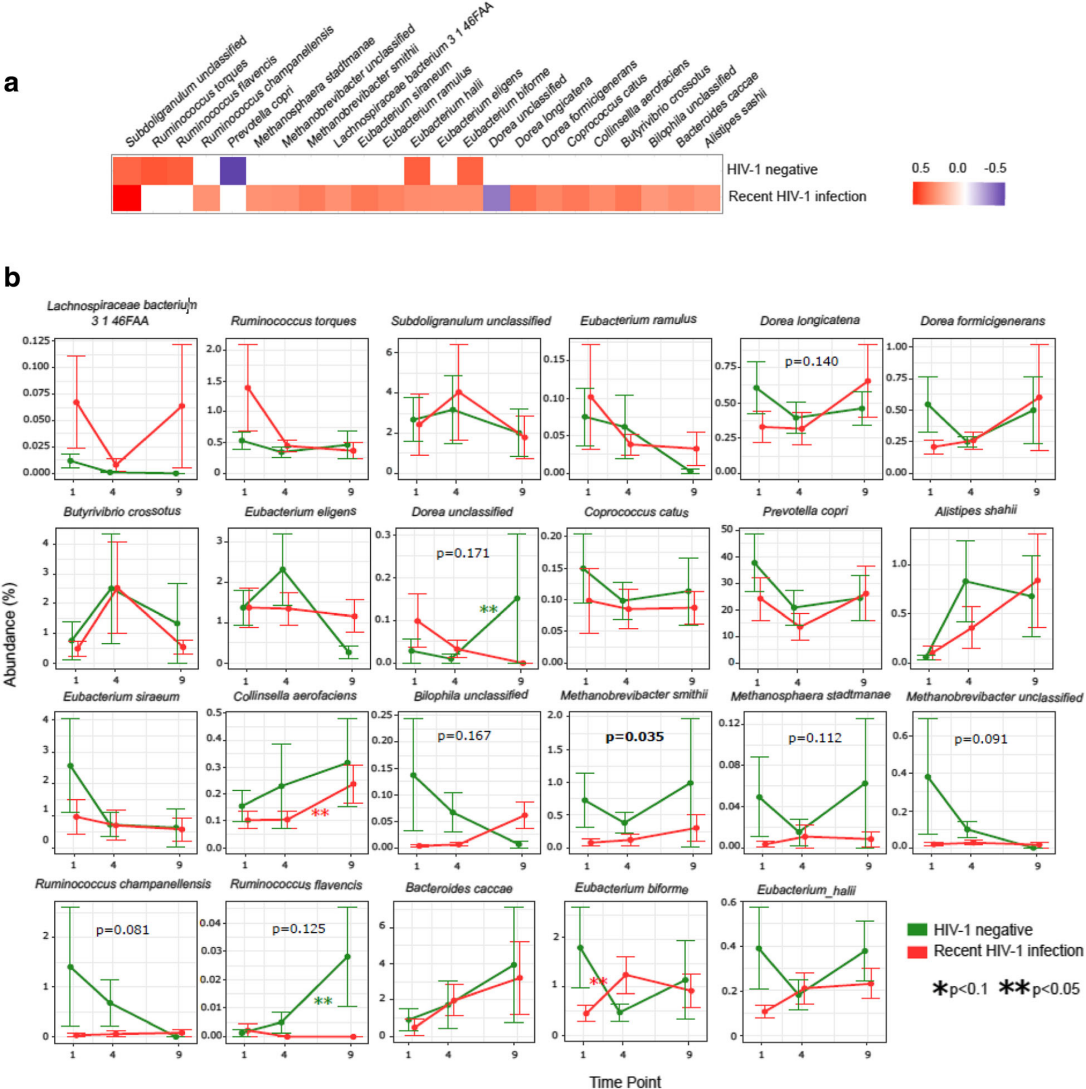
Evolution of richness-associated microbial species in recently HIV-1-infected vs. HIV-negative subjects using shotgun metagenomics. **a** Microbial species significantly (*p* < 0.05) associated with microbial gene richness in both study groups, Spearman’s correlation rho values (red is for positive, blue is for negative correlation). **b** Relative abundance of richness-associated bacterial species over time (months 1, 4, and 9). *p* values within each box compare month 1 to 4 area under the curves of subjects with recent HIV-1 infection (RHI) versus HIV-negative individuals (NEG). Asterisks show slope values significantly different from 0 in linear mixed models for each bacteria and study group

Like in previous European populations, microbial species positively correlated with higher gene richness included *Subdoligranulum* spp., methanogenic archaea, several butyrate producers from the *Ruminococcus*, *Dorea* and *Eubacterium* genera, and *Butyrivibrio crossotus* (Fig. 5a). *Prevotella copri* was negatively correlated with gene richness in this study, which is consistent with the compositional nature of the microbiome in this *Prevotella*-rich African population [33, 34]. An unclassified species from genus *Dorea* was the unique taxa negatively correlated to gene richness in the RHI group.

In a longitudinal evaluation, several microbial species associated with gene richness were depleted in RHI compared to HIV-negative individuals (Fig. 5b). In general, such depletion occurred already at the first timepoint and in some cases (i.e., methanogenic archaea, *Ruminococcus champanellensis*, *R. flavencis*) did not recover during the available follow-up. Linear mixed models only identified significant increases in *Eubacterium biforme* and *Collinsella aerofaciens* in RHI and increases in *Dorea* unclassified and *Ruminococcus flavencis* in HIV-negative subjects. Consistent with previous reports [23], subjects with RHI had higher counts of bacterial antioxidant enzymes like catalase, bacterioferritin, glucose-6-phosphate dehydrogenase, or peroxiredoxin, although this shotgun analysis was underpowered to identify more granular differences (Additiona1 file 1: Figure S6).

#### Fecal virus shedding

HIV-1 infection was associated with increased fecal shedding of *Adenovirus* measured by RT-PCR. This was observed in RHI (53% of subjects) but also in chronically HIV-1-infected individuals, regardless of whether they were ART-naïve (51%) or received ART (44%) (all *p* values < 0.05, relative to 20% of HIV-1-negative subjects) (Table 2). Conversely, increased shedding of *Cytomegalovirus* and *Enterovirus* was only observed in CHI_noART subjects (6% and 21%, respectively), relative to NEG (2% and 4%, respectively) (all *p* values < 0.05). *Human herpes viruses 6A*, *6B*, and *8* were not detected in any individual, despite using proper extraction and positive PCR controls.

**Table 2:** Prevalence of fecal virus shedding

	Recent HIV-1 infection	Chronic HIV-1 infection		HIV-negative
		ART-naive	On ART	
Adenovirus	26/49 (53.2%)**	36/71 (50.7%)**	12/27(44.4%)**	11/55 (20.0%)
Cytomegalovirus	3/49 (6.1%)	4/71 (5.6%)*	1/27 (3.7%)	1/55 (1.8%)
Enterovirus	1/43 (2.4%)	4/19 (21.1%)*	1/25 (4.0%)	2/45 (4.4%)
Human herpes virus 6A, 6B, and 8	0/49 (0%)	0/71 (0%)	0/71 (0%)	0/55 (0%)

Numbers (percent) of subjects with detectable virus in feces by qualitative commercial real-time PCR. To avoid ascertainment bias due to different follow-up between groups, only the first fecal sample available for testing is used for comparison

*p < 0.1, **p < 0.05; Fisher’s pairwise comparisons relative to HIV-1 negative

Using 16S rRNA gene sequencing to assess bacterial taxa, fecal *Adenovirus* shedding was linked to increased relative abundance of an unclassified *Mogibacteriaceae* genus and lower relative abundance of an unclassified *Erysipelotrichaceae* genus. *Cytomegalovirus* shedding was only linked to lower abundance of family *Clostridiaceae*. *Enterovirus* shedding was linked to lower abundances of *Parabacteroides*, *Akkermansia*, and an unclassified genus from the bacterial family *Rikenellaceae* as well as to higher abundance of an unclassified genus from the family *Elusimicrobiaceae* (Additional file 1: Figure S7)*.*

*Adenovirus* shedding was significantly associated with higher FABP2 and IgG ASCA levels in blood and to lower levels of IL10 and IgG2 (Additional file 1: Figure S8). *Enterovirus* detection in feces was positively associated to higher levels of IL13, IL8, FABP2, CXCL16, CD40L, and EGF in blood and zonulin levels in feces and to lower levels of MIG and sCD1643 in blood. *Cytomegalovirus* shedding was associated to higher IgA and MCP1 and lower levels of IL15 in blood.

## Discussion

This study shows that acute HIV-1 infection is followed by increased fecal adenovirus shedding, which persists during chronic HIV-1 infection and does not resolve with ART. In genus-level analyses, HIV-1 infection is followed by transient changes in the gut microbial richness and composition, which are not HIV-specific. An HIV-1-associated microbiome signature—featuring depletion of *Akkermansia*, *Anaerovibrio*, *Bifidobacterium*, and *Clostridium—*only becomes evident in chronically HIV-1-infected subjects. Finer species-level shotgun metagenomics analyses suggest an early and sustained depletion of methanogenic archaea and several fiber-consuming butyrate-producers following HIV-1 infection.

By using RT-PCR, we purposely performed a targeted analysis of a discrete set of eukaryotic viruses with known pathogenic potential, rather than a comprehensive shotgun virome analysis. Most of the gut virome is composed by bacteriophages of uncertain pathogenicity, and previous analyses using shotgun sequencing found no relationship between the gut bacteriophage composition and HIV-1 [25] or SIV infection [35].

Our findings, however, are consistent with previous studies in non-human primates, where SIV infection was associated with expansion of the enteric virome with minimal changes in the bacteriome [26, 35]. Using RT-PCR, we observed that fecal *Adenovirus* shedding not only was present in more than half of RHI subjects, but also in subjects with chronic HIV-1 infection, being rare in HIV-negative controls. In contrast, fecal CMV and *Enterovirus* shedding was mostly observed in untreated chronically HIV-1-infected individuals, suggesting that CMV shedding might require more prolonged immune dysregulation to occur. Several intestinal eukaryotic viral families including *Adenovirus*, CMV, and *Enteroviruses* promote immune activation in the context of HIV-1/SIV infection [26]. Indeed, *Adenovirus*, *Enterovirus*, and CMV fecal shedding were linked to several immune markers of mucus alteration (FABP2, IgG ASCA, IgA, and MCP1), epithelial disruption (zonulin and EGF), and T cell proliferation, activation, and infiltration (CXCL16, IL10, and CD40L), hence reinforcing their role in the inflammatory response to HIV-1 infection. The persistence of fecal *Adenovirus* shedding in ART-treated subjects is worrying for clinicians, because it might be a relentless source of immune activation difficult to curtail with available treatments. Of note, HHV-6 and HHV-8 were not related at all with gut microbiome changes following HIV-1 infection. No differences in eukaryotic viruses fecal shedding were observed between males and females.

An intrinsic limitation of our study design is that we did not prospectively screen HIV-negative subjects at risk for HIV-1 infection. Thus, we missed the very early events occurring between HIV-1 infection and diagnosis. Bearing this limitation in mind, the “increase—plateau” microbial richness dynamics observed in RHI and NEG groups could be explained by a return to baseline values after a transient richness depletion due to an external insult. Microbiome richness kinetics in HIV-infected subjects mirrored that of CD4^+^ T cell counts and were inversed to HIV-1-RNA kinetics. However, the observation of similar microbiome dynamics in HIV-negative individuals shows that such changes were not HIV-1-specific. Of note, most HIV-negative controls in our study were seeking medical care due to fever and/or mononucleosis-like symptoms when they were recruited into the study, and this possibly affected microbial richness as well. More sensitive shotgun metagenomics analyses suggested that early microbial gene richness reductions resolved earlier in HIV-negative individuals than in HIV-1-infected subjects, but larger studies are needed to confirm this observation. Previous studies have linked lower microbial gene richness to lower nadir CD4+ T cell counts [32], but such variable was not collected in our study. Nonetheless, we did not find any significant correlation between gene richness and most recent CD4+ T cell counts, neither for the RHI and NEG groups.

The gut microbial composition also experienced transient longitudinal changes, with reductions in bacterial clusters associated with TNF-mediated innate immune responses and increases in certain butyrate-producing bacteria like *Odoribacter*. Increased abundance of potentially pathogenic *Proteobacteria* has been reported in chronic HIV-1 infected individuals [5, 18, 28, 29] and non-human primates with acute SIV infection [24], and members of the *Enterobacteriaceae* family have been associated to immune activation and inflammation in vivo and ex vivo [5, 28]. However, none of the gut microbiome changes observed in our study was clear enough to be considered HIV-specific.

This study was performed in a *Prevotella*-rich African population. The lack of *Prevotella* to *Bacteroides* shifts or changes in the *Firmicutes*:*Bacteroidetes* ratio in longitudinal follow-up provided further evidence against the previously reported association between these parameters and HIV-1 infection [20, 21, 36, 37]. We did not collect information on same-sex relationships, a potential confounder in microbiome studies [22], because this was a sensitive issue in local culture and all men reported heterosexual sex in previous questionnaires. There is extensive data demonstrating *Prevotella* predominance in African populations [33, 34], so the effect of same-sex intercourse on the gut microbiome would have been hard to discern in this study. Although significant differences in diet have been described between African and European populations and its role on the gut flora [34], collecting information on diet was not included in the original project design. Hence, we were unable to test the effect of diet in relation to the gut microbiota in this study. An interesting collateral, previously unexplored aspect of our study is that the microbial species associated with gene richness in this African population were almost identical to those described in European populations [13, 23].

The only statistically significant association between the gut microbiome composition and HIV-1 infection was a reduction in *Akkermansia*, *Anaerovibrio*, *Bifidobacterium*, and *Clostridium* in chronically HIV-1-infected subjects relative to HIV-negative individuals. Interestingly, *Bifidobacterium* and *Clostridium* have anti-inflammatory effects, and have been previously associated with HIV-1 infection [38–40], so reductions in these genera might contribute to chronic inflammation in HIV-1-infected subjects. *Akkermansia* plays a key role in improved human fat and sugar metabolism [41] and promotes CD8+ T cell-mediated immune responses to anti-cancer immune modulators like PD-1 and CTLA4 agonists [42]. Defects in *Akkermansia* might thus be related to HIV-1-associated metabolic disorders as well as to CD8+ T cell anergy. In our study, *Akkermansia* and *Clostridiaceae* abundance were negatively correlated with markers of microbial translocation (sCD14), inflammation (IP10), and gut integrity (fecal calprotectin). Interestingly, ART initiation was not associated with improved abundance of such microbes. However, proper longitudinal studies are still needed to assess the impact of ART on the microbiome.

To our knowledge, this is the most comprehensive prospective study investigating the longitudinal impact of HIV-1 infection in the gut microbiome in a hyperendemic area such as the Manhiça district in Mozambique, which was previously reported to have a 40% HIV-1 prevalence in adult population [43]. It also incorporates cross-sectional comparisons with chronic HIV-infected subjects, which have been highly informative of the longer-term effects of HIV-1 infection in the gut microbiome. However, this study has a number of limitations.

First, HIV-1-infected subjects were followed more often than HIV-negative individuals and there was a significant amount of loss to follow-up, particularly among HIV-negative subjects. This could have led to ascertainment biases and affected our ability to detect further associations. Following healthy individuals in clinical trials for prolonged periods of time in resource-limited settings is very difficult due to a variety of structural constraints [44]. Linear mixed models can partially account for information loss, but it is evident that lost to follow-up adds uncertainty to our evaluations and might have affected our observations. A second limitation is that, due to the study design, we missed the hyperacute phase of HIV infection. Such phases can only be studied either through very large and costly HIV-negative prospective screening programs, which are out of our reach, or through controlled experiments in non-human primates, which might not always reflect events in humans. A third limitation is that most HIV-negative subjects were not completely healthy when they were recruited into the study. However, this helped us recognize that the transient changes observed in the gut microbiome of HIV-1-infected subjects were not, in fact, HIV-1-specific. Fourth, shotgun metagenomic data was only available for a subset of prospectively followed HIV-1-infected and HIV-negative subjects and was not available for chronic HIV-1-infected subjects. Finally, we did not perform a formal comprehensive virome analysis but, instead, focused on specific viruses with known pathogenic potential. As mentioned before, this strategy has advantages and limitations.

## Conclusions

In conclusion, our study shows that HIV-1 infection is followed by increased fecal *Adenovirus* shedding and by transient, non-HIV-specific changes in the gut bacteriome. Despite early resilience to change, an HIV-1-specific signature in the gut microbiome including depletion of *Akkermansia*, *Anaerovibrio*, *Bifidobacterium*, and *Clostridium*—previously associated with chronic inflammation, CD8+ T cell anergy, and metabolic disorders—can be identified in chronically HIV-1-infected subjects. Longitudinal studies addressing the ability of ART to prevent and/or recover such changes are needed.

## Methods

### Experimental models and subjects’ details

#### Human subjects

This was a sub-study of a prospective observational cohort study (The GAMA cohort Study) [30, 45] originally designed to identify systemic and gastrointestinal inflammation biomarkers of recent HIV-1 infection. The study population was enrolled between April 2013 and May 2014 at the Manhiça District Hospital (MDH), district of Manhiça, Southern Mozambique. The study was approved by local institutional review boards at the Barcelona Clinic Hospital (2011/6264) and by the Ministry of Health of Mozambique (461/CNBS/12). Written informed consent was obtained from patients before participation.

This microbiome sub-study had two components (Additional file 1: Figure S1):

- First, we conducted a prospective observational cohort study to characterize the longitudinal changes occurring in the fecal microbiome of individuals with recent HIV-1 infection (RHI) and HIV-negative controls (NEG) during at least 9 months after their initial HIV-1 assessment. Adults older than 18 years, who were residents of the established district surveillance system study area and who presented to the outpatient clinic of the MDH for nonspecific febrile symptoms or voluntary HIV counseling and testing were invited to participate in the study.
- Second, data from this longitudinal cohort was compared to a single evaluation from chronically HIV-1-infected subjects followed at the outpatient HIV-1 clinic at the MDH, who were receiving antiretroviral therapy (CHI_ART) or not (CHI_noART). For this cross-sectional comparison, we divided the longitudinal follow-up of RHI in two periods: before and after the first 6 months of follow-up (RHI≤6 and RHI > 6), and only included data from the first sample available from each period, as well as the first sample available from each HIV-1 negative subject (NEG).

Pregnant women were not allowed to enter the study.

#### Diagnosis of recent HIV-1 Infection

Blood was collected by finger prick for HIV rapid antibody testing with Determine HIV 1/2 (Abbott Laboratories, Chicago, IL, USA). Positive results were confirmed with a more specific Uni-Gold rapid test (Trinity Biotech Co., Wicklow, Ireland). Individuals with positive HIV-1 serology in both rapid tests were not eligible for enrollment into the study and were referred for clinical management. Recent HIV infection (RHI) was defined as a negative or indeterminate rapid test serology (first test negative or first test positive and second test negative) and positive HIV-1 viremia by reverse transcriptase–polymerase chain reaction on frozen plasma (Abbott Real-Time HIV-1 Assay, limit of detection 150 copies/mL). HIV-1 RNA testing was performed by applying a multilevel pooling scheme of 10 samples/pool as described before [46]. A subgroup of HIV-negative (NEG) time-matched controls was selected by computer randomization, which were also prospectively followed and served as controls for RHI.

#### Study follow-up

Subjects with RHI were seen at months 1, 2, 3, 4, 6, 9, 12, 15, and 18 after study enrollment, whereas HIV-negative controls were seen at months 1, 4, and 9. This was considered the only follow-up realistically feasible in otherwise healthy subjects in the Mozambican setting. Medical consultation and HIV counseling were provided at each medical visit. Study participants received antibiotic treatment as required according to their clinical status. They also began ART when needed, according to the Mozambican ART guidelines applicable at the time of the study (i.e., when CD4+ T cell counts were lower than 250 cells/mm^3^ or clinical complications requiring ART initiation occurred). If subjects had to start antibiotics or ART, they provided a last fecal sample and the study follow-up was immediately terminated. Similarly, the study follow-up was interrupted if women became pregnant. Only one clinical and analytical assessment was performed in chronically HIV-1-infected adults. In this pilot exploratory study, no formal sample size calculation was performed.

### Method details

#### Demographic and clinical data

Demographic and clinical data were collected in a specific questionnaire which included the study group to which the patient belonged (RHI, NEG, CHI_ART, and CHI_noART); the timepoint of follow-up in months; the participant’s gender, age, weight, and height; his/her CD4^+^ and CD8^+^ T cell counts; the CD4^+^/CD8^+^ ratio; HIV-1 RNA levels; hemogram; body temperature; whether fever was reported or not within the previous 24 h of sample collection; whether the patient reported or not diarrhea the week before sample collection and its severity; and information on pregnancy, antiretroviral therapy, and antibiotic use.

The dataset also included information on a malaria optic microscopic assessment performed the day of sample collection, whether the participant reported malaria within the previous month of sample collection, the presence of occult blood in feces, and the result of microbiological tests in feces, including *Giardia* spp., *Cryptosporidium* spp., *Entamoeba* spp., *Clostridium difficile* toxin, as well as serologies for hepatitis B and C, syphilis and *Strongyloides stercoralis*.

#### 16S rRNA gene sequencing: DNA extraction, amplicon generation and sequencing

Fecal DNA was extracted using the PowerSoil DNA Extraction Kit (MO BIO Laboratories, Carlsbad, CA, USA). The V3–V4 variable regions from the 16S rRNA gene (amplicon size expected ~ 460 bp) were PCR-amplified using the MiSeq rRNA Amplicon Sequencing protocol and primers 16S_F 5′-(TCG TCG GCA GCG TCA GAT GTG TAT AAG AGA CAG CCT ACG GGN GGC WGC AG)-3′ and 16S_R 5′-(GTC TCG TGG GCT CGG AGA TGT GTA TAA GAG ACA GGA CTA CHV GGG TAT CTA ATC C)-3′. Amplification was performed in 25 reactions containing 2.5 μl of non-diluted DNA template, 0.8 μl of each primer at 10 μM, 8.4 μl of DNA and RNA-free water, and 12.5 μl of KAPA HiFi HotStart Ready Mix (containing KAPA HiFi HotStart DNA Polymerase, buffer, MgCl2, and dNTPs, KAPA Biosystems Inc., Wilmington, MA, USA). Thermal cycling conditions consisted of an initial denaturation step (5 min at 95 °C), followed by 30 cycles of denaturation (20 s at 98 °C), annealing (15 s at 69 °C), and extension (15 s at 72 °C). These were followed by a final extension step of 1 min at 72 °C. Once the desired amplicon was confirmed in 1% agarose gel electrophoresis, the amplified DNA was stored at − 30 °C until library preparation. Amplified DNA templates were cleaned from non-DNA molecules and Illumina sequencing adapters, and dual indices were attached using the Nextera XT Index Kit (Illumina, Inc.) followed by the corresponding PCR amplification program as described in the MiSeq 16S rRNA gene Amplicon Sequencing protocol. After a second round of cleanup, amplicons were quantified using the Quant-iT™ PicoGreen® dsDNA Assay Kit (Invitrogen, Carlsbad, MA, USA) and Nanodrop 1000 (Thermo Scientific, DE, USA) and diluted in equimolar concentrations (T4 nM) for further pooling. Sequencing was performed on an Illumina MiSeq^TM^ platform (Illumina, Inc.) according to the manufacturer’s specifications to generate paired-end reads of 300 base length in each direction.

#### Whole genome sequencing (WGS): library preparation and sequencing

In addition to 16S rRNA gene sequencing, a subset of 54 samples was evaluated using shotgun fecal metagenomic sequencing. Subjects chosen for shotgun sequencing analysis had fecal samples available for testing at least at months 1 and 4. If available, month 9 from these same subjects was also sequenced. Extracted, non-diluted DNA was fragmented with the Nextera-XT Illumina kit (Illumina, Inc.) following the manufacturer’s instructions. One library of approximately 300 bp clone insert sizes was constructed per sample. Samples were sequenced in an Illumina HiSeq sequencer (Illumina, Inc.).

#### 16S rRNA gene sequence analysis

Raw Illumina MiSeq sequences were filtered using a minimum quality threshold of Q20 in at least 50% of the bases and a minimum sequence average quality of Q20. The 1.9.1 version of Qiime (Quantitative Insights Into Microbial Ecology) software pipeline [47] was used for taxonomic classification of 16S rRNA gene sequences contained in each sample, using GreenGenes 13.8 as the reference database [48]. The first step was to join forward and reverse reads per each sample using the join_paired_ends.py script. The maximum percent differences accepted in the overlapping region were set to 15. Reads were then filtered to contain a maximum of 2 ambiguous bases (*N*) using the split_libraries_fastq.py script. An open-reference OTU picking approach was used to cluster reads at 97% sequence similarity and construct operational taxonomic units (OTUs). Briefly, in this pick_open_reference_otus.py approach, reads are clustered against a reference sequence collection and reads that do not hit any reference sequence are subsequently clustered using a de novo clustering approach. We then used the ChimeraSlayer [49] method in the parallel_identify_chimeric_seqs.py script to identify chimeric sequences using a PyNast reference from the GreenGenes 13.8 database. Finally, make_otu_table.py, filter_alignment.py and make_phylogeny.py scripts were used to create final biom and phylogenetic tree files.

#### Metagenomic sequence analysis

Raw HiSeq sequences were filtered using Trimmomatic [50]. Nextera adapters were removed, and reads were trimmed enabling a minimum quality of Q30, a global minimum length of 100 bp and using a sliding window set at a minimum quality of Q20 for each 30-bp-long consecutive segments. Human contamination was removed by mapping filtered sequences against the human genome and removing reads with an alignment quality above Q20. Paired filtered reads were used for taxonomic characterization of microbial communities using MetaPhlan2 software with the default parameters [51]. Entire sets of filtered reads were aligned using Bowtie2 to the integrated reference catalog of the human gut microbiome (IGC) (http://meta.genomics.cn/meta/home) [52]. Resulting alignments were then filtered with Samtools [53] so that only reads with an alignment quality above Q20 were kept. Additionally, chimeric and secondary alignments were also removed. Eventually, a subset of six million aligned reads was created per sample alignment to ensure that results of gene KEGG (http://www.genome.jp/kegg/) and metabolic pathway richness were comparable between samples.

#### Detection of fecal virus shedding by qualitative RT-PCR

We selectively evaluated the presence of viruses with human pathogenic potential—i.e., *Adenovirus* (ADV), *Cytomegalovirus* (CMV), *Human Herpesvirus* (HHV) 6A and 6B, and 8 and *Enterovirus* (ETV)—in cryopreserved stool DNA and RNA aliquots using commercial RT-PCR kits and following the manufacturer’s instructions, including adjustment of quantification using a sensitivity control at 1 copy/μl. For ADV, the Adenovirus R-gene® kit (BioMérieux, Marcy-l’Étoile, France) was used. For CMV, HHV 6A, 6B, and 8, specific probe/primer mixes for RT-PCR assays (Virusys Corporation, TaneyTown, MD, USA) were used in combination with the TaqMan® Gene Expression Master Mix (Thermo Scientific, DE, USA) at 1/20 dilution. For ETV, fecal RNA was first extracted from RNAlater®-cryopreserved fecal samples using the Stool total RNA Purification Kit (Norgen Biotek, Corporation, Thorold, Canada). Non-diluted fresh RNA aliquots were directly used to perform qualitative RT-PCR testing of enteroviruses using the Enterovirus R-gene® Kit (BioMérieux, Marcy-l’Étoile, France).

#### Cytokine quantification in plasma

A total number of 51 cytokines were quantified in plasma samples including general markers of inflammation, cell death and growth factors, angiogenesis, monocyte function and mobility, T and B cell function, neutrophil and eosinophil activation, enterocyte damage, intestinal permeability, and microbial translocation. Detection of IL-1R antagonist, MCP-1, GCSF, IFN gamma, IL-12, IL-13, IL-7, VEGF, MIG, RANTES, Eotaxin, MIP1 beta, IP-10, IL-2R, IFN alpha, IL-15, GMCSF, TNF alpha, IL-1β, IL-2, IL-4, IL-5, IL-6, IL-10, MIP1 alpha, IL-17, IL-8, and EGF were determined by using Human Cytokine Magnetic 30-Plex panel (Invitrogen). Detection of CD40 ligand and IL21 was performed by using the Bio-Plex Pro Human Th17 cytokine assay (Bio-Rad). Detection of BAFF, CD27, and TNFR2 was performed by using a Human Magnetic Luminex Screening Assay (R&D). Detection of sCD14, LBP, FABP 2, CRP, sCD163, CXCL16, sCD23, B7H1, PD ligand 2, TRAIL, Fas ligand, TGF beta 1 (R&D); IgG ASCA and IgA ASCA (Orgentec); EndoCab IgG and EndoCab IgA (Hycult biotech); and zonulin (Immundiagnostik) was assessed by enzyme-linked immunosorbent assays (ELISA) procedures. All quantifications were performed according to the manufacturer’s instructions. Overflow and under limit of detection values were validated for every analyte as the double and the half of the detection limit, respectively.

Additional quantification of general antibody types and subtypes was also performed by ELISA using the following antibodies: polyclonal affinity pure goat anti-human IgA antibody α-Chain Specific (Jackson Immunoresearch), mouse anti-human IgM antibody (clone G20-127) (BD Pharmingen), mouse anti-human IgG1 antibody (clone HP6069) (Life technology), mouse anti-human IgG2 antibody (clone G18-21) (BD Pharmingen™), mouse anti-human IgG3 Hinge (clone HP6050 ) (SouthernBiotech), mouse anti-human IgG4 antibody (clone G17-4) (BD Pharmingen™), and polyclonal affinipure F(ab')_2_ fragment goat anti-human IgG, Fcγ fragment specific.

#### Cytokine quantification in feces

Determinations in stool samples were performed by ELISA commercial assays according to the manufacturer’s instructions for calprotectin, PMN-elastase, zonulin, EDN/EPX, HBD2, secretory IgA1, α1–antitrypsin, S100A12, and claudin (Immundiagnostik, Bensheim, Germany); lactoferrin, ASCA, and pANCA (TechLab, Blacksburg, USA).

### Quantification and statistical analyses

#### Ecological analysis of gut microbiota

Bacterial taxonomic richness (observed richness and the numeric richness estimators Chao1 and ACE) as well as diversity/evenness measurements (Shannon and Simpson indices) were calculated using Vegan [54] and BiodiversityR [55] R packages, correspondingly. We modified the Simpson index’s formula so that it was computed as 1/(1-Simpson concentration index). OTUs present uniquely in one sample were filtered out to reduce noise. For each of the samples, a subset of 5000 counts was randomly selected using the *rrarefy* function from Vegan, as representative of the entire sample sequence set. Samples with less than 5000 counts were not considered for this ecological analysis.

#### Analysis of gut microbiota composition

All reads available were used to assess bacterial composition. OTUs present in less than 10 counts in 10% of samples were filtered out. Then, OTU counts were collapsed to different taxonomic levels using Phyloseq [56] R package and the corresponding relative abundance values were calculated.

Differences in overall microbiota composition were evaluated using Non-Metric Multidimensional Scaling (NMDS) ordination analysis on Bray-Curtis, Euclidean, weighted UniFrac and unweighted UniFrac distances as available in Phyloseq [56] and Vegan [54] R packages. Permutational analysis of variance using Bray-Curtis distances as implemented in *adonis* function from the Vegan R package was used to test the influence of different metadata variables on microbiota composition similarity measures. The PAM algorithm implemented in Cluster [57] R package was used to test for the existence of clusters of samples based on Bray-Curtis distance matrices computed among samples.

Clustering of bacterial genera into co-abundance groups was performed using *hclust* algorithm in R stats package with ward.D2 methodology on an Euclidean distance matrix computed from a relative abundance table of bacterial genera across groups. For each genus and group, we calculated the mean value of all previously scaled individual abundance percentages (force mean = 0 and sd = 1). A phylogenetic distance threshold of 0.8 allowed us to identify 7 different bacterial clusters on the resulting dendrogram.

The LEfSe algorithm [58] was used to describe which bacterial genera were significantly enriched or depleted in association with positive and negative detection of *Adenovirus*, *Enterovirus* and *Cytomegalovirus* in feces. Comparisons were done using both the soft and the strict statistical criterion regarding eukaryotic viral detection and HIV-1 infection status. To increase sample size in tested groups for the latter variable, samples from RHI, CHI_ART and CHI_noART groups were categorized as “Positive” meanwhile the samples form NEG remained as “Negative”.

#### Analysis on gut microbiota’s gene richness and metabolic functions

Filtered metagenomic sequences were mapped against the Integrated Gene Catalog (IGC) [52] using the bwa software [59]. Unique alignments with a minimum quality of Q20 were selected for subsequent analyses. To be able to compare the microbial gene content across samples we used a downsampling size of six million aligned sequences.

Gene richness was measured as the total number of different genes present in the sample regardless of their abundance and length. A minimum of one filtered mapped sequence was set to consider the presence of a gene. The copy number of each gene was estimated by dividing the total reads mapping to a gene divided by the gene’s length. A gene’s relative abundance was measured as its copy number divided by the sum of the total gene copies in the sample. As in previous studies [13], gene richness followed a bimodal distribution in a probability density function. The local minimum between the two modes was considered the threshold value to classify individuals in two groups according to their microbial gene richness content: high gene counts (HGC) and low gene counts (LGC).

#### Microbial functional profile

Genes found in shotgun metagenomes downsampled at 6 M sequences were associated to one or multiple KEGG categories according to the IGC reference database [52]. To measure KEGG abundances, all genes associated to the same KEGG category were collapsed and their copy numbers were summed. If a gene was associated to more than one KEGG category, all categories were considered separately. The relative abundance of KEGG categories was measured as in the relative gene abundance approach detailed above. To analyze the metabolic pathways represented in all samples, we fed the HUMAnN pipeline [60] with the KEGG copy number table. The HUMAnN output included the relative abundances of metabolic modules and pathways for each subject. KEGG functions that were not associated to prokaryotic or archaeal metabolism according to KEGG catalog information [61] were manually filtered out, to ensure that only microbial functions were analyzed.

#### Fecal virus shedding analysis

Results derived from RT-PCR testing were used as presence/absence of viral DNA/RNA in feces. Comparison of number of individuals with virus in feces among groups at study entry was performed using Fisher’s exact statistical test. To summarize results from tested samples from the same individual at different time points to a single value per individual, we used two different criteria: 1) if the first sample available from that given individual was considered positive, that subject was positive, 2) if that given individual had at least 1 sample testing positive along its follow-up, that subject was considered positive.

#### Statistical analysis

Longitudinal changes in the RHI and NEG groups were analyzed using linear mixed models. After visual inspection of raw data, we allowed for a biphasic modeling of such longitudinal changes, allowing for an inflection point at month 6 for RHI—as this is the accepted duration of recent HIV-1 infection [62], and at 4 months for NEG subjects, because the parental GAMA study chose to follow subjects at 1, 4, and 9 months and no 6-month data was available. Linear mixed models from the first phase were fitted using a random intercept, whereas the last point from the first LMM was used as the intercept for the second LMM. Separate statistics were then performed for each LMM slope. In both cases, *p* values indicated that the slope was significantly different from 0.

For the cross-sectional comparisons, intra-patient comparisons at different time points were tested using paired Wilcoxon Rank sum test, whereas comparisons between two groups were tested using non-paired Wilcoxon rank sum test. Comparisons between multiple groups were statistically tested using Kruskal–Wallis and post hoc pairwise tests corrected for multiple comparisons using Benjamini–Hochberg or false discovery rate as needed. The Spearman’s correlation test was used to test relation between numerical variables.

## Additional file

Additional file 1: **Table S1.** Study participant’s flow and samples available for testing. **Figure S1.** Diagram of study design with the prospective longitudinal and the cross-sectional components. **Figure S2.** Longitudinal evolution of CD4+ and CD8+ T cell counts and HIV-1 RNA levels. **Figure S3.** Changes in observed richness and Shannon’s diversity using shotgun sequencing. **Figure S4.** Ordination plot of cross-sectional sample dataset using gut microbiota composition. **Figure S5.** Beta-diversity of the fecal microbiomes during the first (RHI < 6) and second 6 months (RHI > 6) following HIV-1 infection. **Figure S6.** Evolution of reactive oxygen species (ROS)-associated enzymes in recently HIV-1-infected vs. HIV-1-negative subjects. **Figure S7.** Differences in bacterial genera relative abundance between adenovirus-, cytomegalovirus- and enterovirus-positive and -negative groups using LEfSe. **Figure S8.** Cytokines and immune measurements in blood and feces differentially abundant between adenovirus, enterovirus, and cytomegalovirus-positive and -negative groups. (DOCX 2327 kb)
