# Supplementary material for: Evolution of the gut microbiome following acute HIV-1 infection
Source: Microbiome. 2019 May 11;7:73. doi: 10.1186/s40168-019-0687-5 (PMC6511141; doi:10.1186/s40168-019-0687-5)

**Table S1. Study participant’s flow and samples available for testing**

| **Months since HIV testing (timepoint)** | **New subjects entering follow-up** | **Subjects under follow-up** | **Subjects subsequently lost to follow-up** | | | | | **Fecal samples available for testing** |
| --- | --- | --- | --- | --- | --- | --- | --- | --- |
|  |  |  | **Total** | **Voluntary patient withdrawal** | **Pregnancy** | **ART initiation** | **Death** |  |
| *HIV-1-infected (n at screening = 95)* | | | | | | | | |
| 1 | 43 | 43 | 14 | 10 | 1 | 2 | 1 | 43 |
| 2 | 2 | 31 | 6 | 3 | 3 | - | - | 27 |
| 3 | 2 | 27 | 8 | 6 | - | 2 | - | 23 |
| 4 | - | 19 | 4 | 3 | - | 1 | - | 18 |
| 6 | 1 | 16 | 3 | 3 | - | - | - | 10 |
| 9 | 1 | 14 | 2 | 2 | - | - | - | 11 |
| 12 | - | 12 | 4 | 4 | - | - | - | 9 |
| 15 | - | 8 | 5 | 5 | - | - | - | 5 |
| 18 | - | 3 | - | - | - | - | - | 3 |
| *HIV-negative (n at screening = 3045)* | | | | | | | | |
| 1 | 54 | 54 | 46 | 46 | - | - | - | 54 |
| 4 | 1 | 9 | 5 | 5 | - | - | - | 9 |
| 9 | - | 4 | - | - | - | - | - | 4 |

Reasons for voluntary patient withdrawal included: loss of interest in the study (n=31), work incompatibilities (n=2), husband prohibition (n=3). Reasons for ART initiation included: CD4^+^ T-cell count ≤350 cells/mm^3^ (n=2), coinfection with Hepatitis B virus (n=1), tuberculosis (n=1) and acute leg paralysis (n=1).

**Figure S1.** Diagram of study design with the prospective longitudinal and the cross-sectional components.


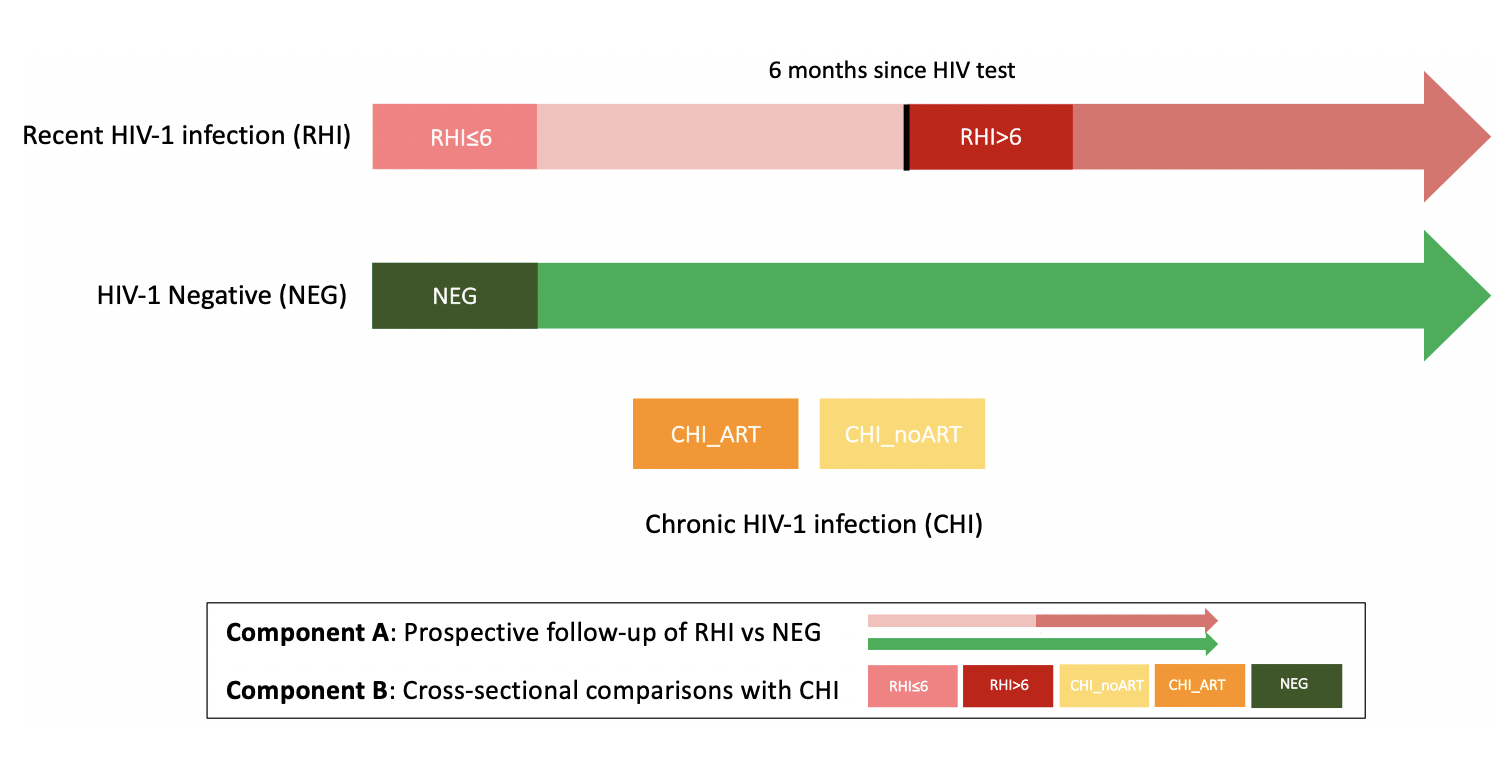


**Figure S2.** **Longitudinal evolution of CD4^+^ and CD8^+^ T-cell counts and HIV-1 RNA levels.**

Subjects with recent HIV-1 infection are colored in red (A) and HIV-negative controls are colored in green (B). Longitudinal changes on immune and viral parameters were obtained using linear mixed models. Statistically significant differences from 0 (flat slope) are shown with asterisks. p-values: *<0.1; **<0.05


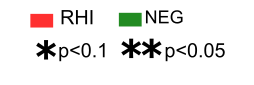


**Figure S3. Changes in observed richness and Shannon’s diversity using shotgun sequencing.**

Microbial genera ecological parameters are shown on the upper panels and species ecological parameters on the lower panels. Such richness and diversity metrics were computed for both, subjects with recent HIV-1 infection (RHI, red) and HIV-negative controls (NEG, green). Median and IQR values per time point and linear mixed models are shown. Statistically significant differences from 0 (flat slope) are shown with asterisks. p-values: *<0.1; **<0.05


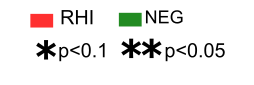


**Figure S4. Ordination plot of cross-sectional sample dataset using gut microbiota composition.**

Non-metric dimensional scaling plots using three different ecologic distances summarizing the 16S-rRNA gene gut microbiome composition at microbial phylum (left) and genera (right) levels of recently HIV-1-infected subjects during the first 6 months (RHI<6) and second 6 months (RHI>6) of follow-up, HIV-negative subjects (NEG), and chronically HIV-1-infected subjects receiving antiretroviral therapy (CHI_ART) or not (CHI_noART). To limit ascertainment bias, only the first microbiome measurement from RHI<6, RHI>6 and NEG was used for comparisons with CHI_ART and CHI_noART.


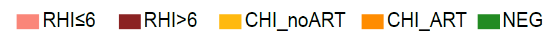


**Figure S5.** **Beta-diversity of the fecal microbiomes during the first (RHI<6) and second 6 months (RHI>6) following HIV-1 infection**.

Using 16S-rRNA gene data with Bray Curtis and Weighted UniFrac ecologic distances, as well as with shotgun data both at microbial genera and species levels. To limit ascertainment bias only the first microbiome measurement available for RHI<6 and RHI>6 was used for comparisons. Statistically significant P-values obtained with the Wilcoxon-paired test are shown.

**Figure S6. Evolution of reactive oxygen species (ROS)-associated enzymes in recently HIV-1-infected vs. HIV-1-negative subjects**.

Relative abundance of ROS-associated enzymes over time (months 1, 4 and 9). P-values within each box compare month 1 to 4 area under the curves of subjects with recent HIV-1 infection (RHI) versus HIV-negative individuals (NEG). Asterisks show slope values significantly different from 0 in linear mixed models for each bacteria and study group.

**Figure S7. Differences in bacterial genera relative abundance between *Adenovirus*-, *Cytomegalovirus*- and *Enterovirus*- positive and negative groups using LEfSe**

Bacterial genera enriched in either the *Enterovirus*, *Adenovirus* or *Cytomegalovirus* positive-groups versus the corresponding negative-groups, regardless of the HIV-1 infection serostatus. Linear discriminative analysis (LDA) scores were obtained using LEfSe on the 16S rRNA gene sequencing dataset. Asterisks indicate which bacterial genera remained differentially abundant between *Enterovirus*, *Adenovirus* or *Cytomegalovirus* infection groups considering also HIV-1 infection status (NEG vs RHI/CHI_ART/CHI_noART) (i.e. soft criteria in LEfSe using viral infection status as class and HIV-1 infection status as subclass).
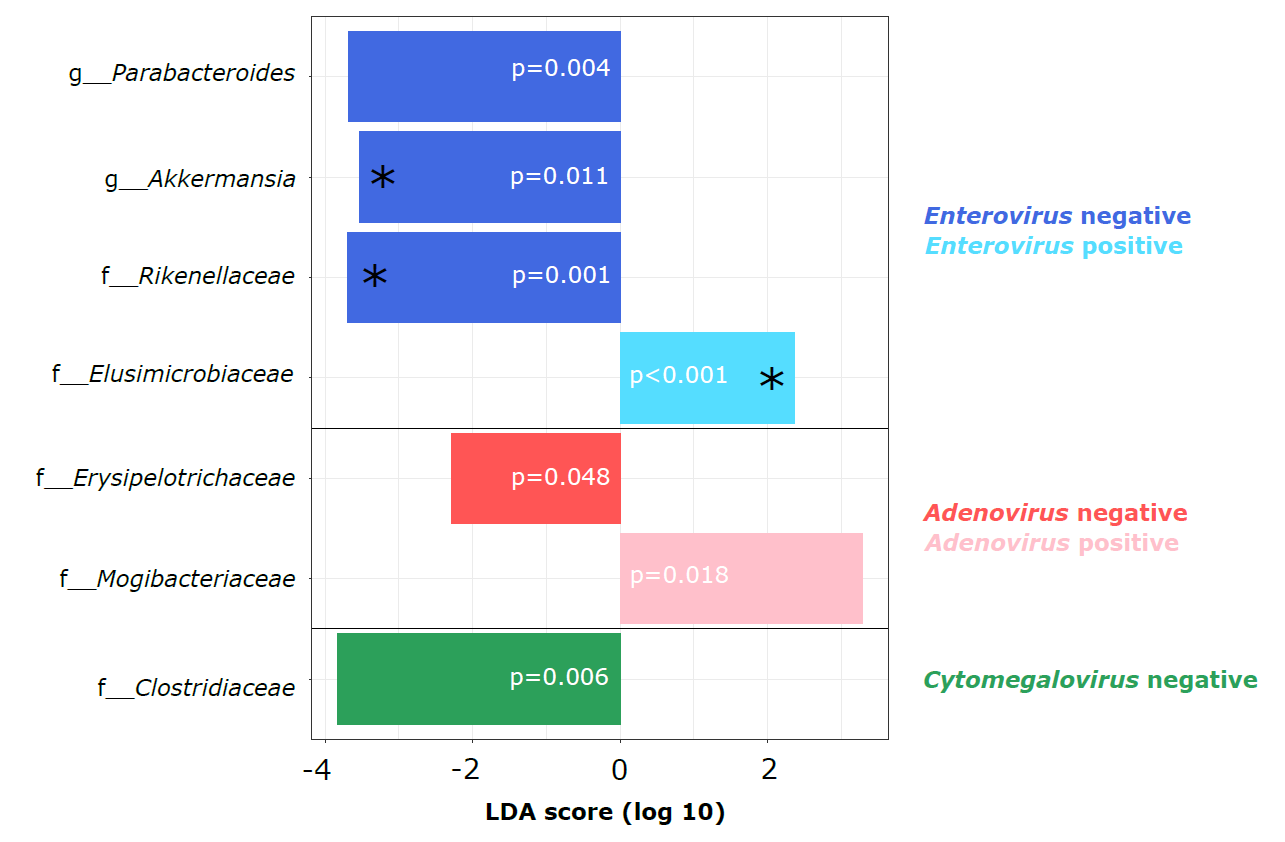


**Figure S8. Cytokines and immune measurements in blood and feces differentially abundant between *Adenovirus, Enterovirus* and *Cytomegalovirus* positive and negative groups.**

Immune measurements differentially abundant between groups reporting positive and negative fecal shedding of *Adenovirus, Enterovirus* and *Cytomegalovirus*. Only immune parameters with an uncorrected significant Wilcoxon Rank Sum Test (p<0.05) between groups are shown. *Zonulin is the only significant parameter measured in feces. All other parameters are measured in blood.


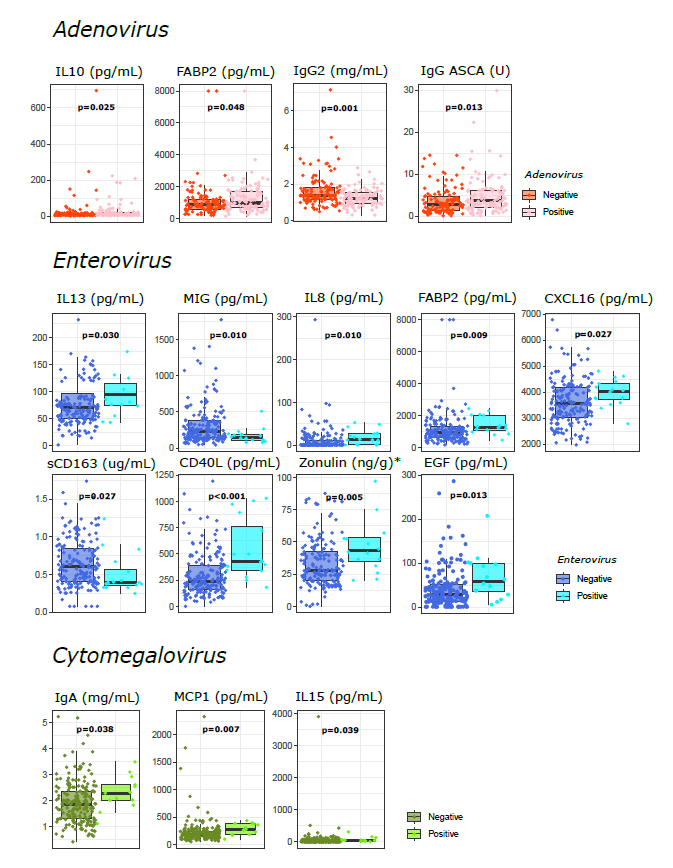

Supplement: Supplementary file 1 — Table S1. Study participant’s flow and samples available for testing. Figure S1. Diagram of study design with the prospective longitudinal and the cross-sectional components. Figure S2. Longitudinal evolution of CD4+ and CD8+ T cell counts and HIV-1 RNA levels. Figure S3. Changes in observed richness and Shannon’s diversity using shotgun sequencing. Figure S4. Ordination plot of cross-sectional sample dataset using gut microbiota composition. Figure S5. Beta-diversity of the fecal microbiomes during the first (RHI < 6) and second 6 months (RHI > 6) following HIV-1 infection. Figure S6. Evolution of reactive oxygen species (ROS)-associated enzymes in recently HIV-1-infected vs. HIV-1-negative subjects. Figure S7. Differences in bacterial genera relative abundance between adenovirus-, cytomegalovirus- and enterovirus-positive and -negative groups using LEfSe. Figure S8. Cytokines and immune measurements in blood and feces differentially abundant between adenovirus, enterovirus, and cytomegalovirus-positive and -negative groups. (DOCX 2327 kb) [file 40168_2019_687_MOESM1_ESM.docx]
